# Use of CRISPR systems in plant genome editing: toward new opportunities in agriculture

**DOI:** 10.1042/ETLS20170085

**Published:** 2017-11-10

**Authors:** Agnès Ricroch, Pauline Clairand, Wendy Harwood

**Affiliations:** 1 AgroParisTech, 16 rue Claude Bernard, Paris F-75231, France; 2 Université de Paris-Sud, Faculté Jean-Monnet, Collège d'Etudes Interdisciplinaires, 54, Boulevard Desgranges, Sceaux F-92330, France; 3 John Innes Centre, Norwich Research Park, Norwich NR4 7UH, U.K.

**Keywords:** agriculture, CRISPR, gRNA, plant genome editing

## Abstract

Initially discovered in bacteria and archaea, CRISPR–Cas9 is an adaptive immune system found in prokaryotes. In 2012, scientists found a way to use it as a genome editing tool. In 2013, its application in plants was successfully achieved. This breakthrough has opened up many new opportunities for researchers, including the opportunity to gain a better understanding of plant biological systems more quickly. The present study reviews agricultural applications related to the use of CRISPR systems in plants from 52 peer-reviewed articles published since 2014. Based on this literature review, the main use of CRISPR systems is to achieve improved yield performance, biofortification, biotic and abiotic stress tolerance, with rice (*Oryza sativa*) being the most studied crop.

## Introduction

CRISPR/Cas systems have proved to be important tools for genome editing in model plants and crops. On the one hand, CRISPR/Cas or tools derived from this technology have permitted rapid and straightforward determination of the function of different coding and non-coding DNA sequences in model plants [1–5]. On the other hand, numerous studies describe applications of CRISPR systems for the development of new traits in crops and could be considered as **proof-of-concept studies**.

The CRISPR/Cas gene-editing system is able to generate heritable, targeted mutations and also to address concerns over the presence of foreign DNA sequences as it can generate **transgene-free plants.** Therefore, it offers advantages in giving precision that was previously not possible and in allowing the induction of mutations without the presence of transgenes in the final plants. Articles focusing on these areas are considered, together with the analysis of the agricultural opportunities offered by the technology within specific geographic areas. We built up the following bibliographic research quest to gather scientific peer-reviewed articles specifically dealing with trait improvement in crops: ‘CRISPR’ OR ‘clustered regularly interspaced short palindromic’ OR ‘cas9’ OR ‘cas 9’ AND (Plant* OR vegetal OR Spermatophyt* OR algae OR Dicot* OR Monocot* OR Legume* OR Cereal* OR crop*). Then, we submitted this research request in databases such as Infodoc, Sciencedirect, BiblioVie, EBSCO, BergeRicrochGMlibrary and Web of Science to perform this systematic literature review. These search terms may not have captured every relevant article; however, those captured clearly identify key trends.

## Induction of heritable targeted mutations and generation of ‘transgene-free’ plants

Regarding economic perspectives and social acceptance of CRISPR/Cas systems, a major concern is the heritability of the gene-induced mutations and the generation of transgene-free plants. Various articles have reported the induction and stable inheritance of single- [6–8] and multiple-targeted mutations [2], studying the T0 plants and T1 and T2 progenies. Generally speaking, the mutants of interest are selected by segregation [9].

Regarding the heritability concern, Pan et al. [8] used a visually interesting tool to demonstrate the inheritance of mutations induced in the *PDS* gene of *Solanum lycopersicum*. *SlPDS* encodes the phytoene desaturase which is a key enzyme in carotenoid biosynthesis. The silencing of this gene causes photobleaching or albino phenotypes. The authors were, thus, able to monitor mutation and inheritance patterns with a visual indicator associated with genotyping and sequencing. They demonstrated that the CRISPR/Cas system can induce heritable mutations in tomato plants (from T0 to T2 generation plants) and that homozygous and biallelic mutants were generated even in the first generation. In addition, a classical study on *Arabidopsis thaliana* provided a general scheme regarding the heritability of mutations induced by CRISPR/Cas using *Agrobacterium*-mediated transformation [10].

To generate transgene-free plants, it is necessary to obtain a stable production of CRISPR/Cas-mutated lines without the presence of the CRISPR DNA expression cassettes in the final mutant plants. This can be achieved in many ways. Most studies using *Agrobacterium*-mediated transformation intend to generate final mutant plants without transferred DNA (T-DNA). As specific primers are used to detect the presence of transgenes encoding CRISPR/Cas components, scientists showed that transgene-free, T2 mutant lines could be obtained by **genetic segregation**: the targeted mutations were stably passed on in transgene-free plants [Table 1, column ‘Transgene-free plants studied (Yes/No)’]. Other studies show how to generate transgene-free plants using alternative delivery methods [11,12]. These methods include ways of introducing the CRISPR components in a transient fashion such that integration is unlikely, for example using protoplast systems [12]. Alternatively, it is possible to avoid the presence of foreign DNA at any stage of the process, therefore avoiding the possibility of foreign DNA insertion. This can be achieved by the introduction of RNA or a ribonucleoprotein complex [13].

**Table 1:** Agricultural applications of the use of CRISPR systems in the 52 articles studied (2014–2017)

Plant species	Application perspectives	Targeted sequence(s)	Molecular functions	Delivery method//main strategy	Transgene-free plants studied (yes/no)	Reference
**BIOTIC STRESS TOLERANCE**						
**Virus stress tolerance**						
***Model plants***						
*Arabidopsis thaliana*	Potyvirus resistance (TuMV)	eIF(iso)4E, member of the eukaryotic translation initiation factor	Recessive resistance alleles against various potyviruses	*Agrobacterium*-mediated transformation with a Cas9/gRNA recombinant plasmid binary vector (floral dipping) // **gene knockout with Cas9/gRNA**	Yes	[9]
*Arabidopsis thaliana* and *Nicotiana benthamiana*	Beet severe curly top virus (BSCTV) tolerance	43 candidate sites in coding or non-coding sequences of the BSCTV genome for transient expression assays and selection of two sites for transgenic lines induction	Virus replication mechanism	*Agrobacterium*-mediated transformation of leaves with Cas9/gRNA expression plasmid vectors // **gene knockout with Cas9/gRNA**	No	[7]
*Nicotiana benthamiana*	Tomato yellow leaf curl virus (TYLCV) resistance	Coding and non-coding sequences of TYLCV	Virus replication mechanism	*Agrobacterium*-mediated transformation of leaves with a TRV RNA replicon for the delivery of gRNAs into Cas9 overexpressing plants // **gene knockout with Cas9/gRNA**	No	[14]
	Virus tolerance	AGO2 gene	Contribution to antiviral immunity (virus-specific antiviral role of AGO2 gene)	*Agrobacterium*-mediated transformation of leaves with Cas9/gRNA expression plasmid vectors // **gene knockout with Cas9/gRNA**	No	[20]
***Crops***						
*Cucumis sativus*	Ipomovirus immunity, tolerance to the Zucchini yellow mosaic virus and Papaya ring spot mosaic virus-W	eIF4E (eukaryotic translation initiation factors 4E)	Host factors for RNA viruses, recessive resistance alleles against viruses	*Agrobacterium*-mediated transformation of cut cotyledons (without embryo) with binary vector containing Cas9/gRNA // **gene knockout with Cas9/gRNA**	Yes	[21]
**Fungus stress tolerance**						
***Crops***						
*Oriza sativa*	Blast (caused by *Magnaporthe oryzae*) tolerance	Ethylene responsive factor ERF transcription factor gene OsERF922	Involved in the modulation of multiple stress tolerance	*Agrobacterium*-mediated transformation of embryogenic calli with Cas9/gRNA-expressing binary vectors // **single and multiplex gene knockout with Cas9/gRNA**	Yes	[22]
*Solanum lycopersicum*	Powdery mildew resistance	SlMlo gene	Major contributor to powdery mildew susceptibility	*Agrobacterium*-mediated transformation of cotyledons with Cas9/gRNA expression plasmid vectors // **gene knockout with Cas9/gRNA**	Yes	[23]
*Triticum aestivum*	Powdery mildew (*Blumeria graminis* f. sp. *Tritici*) resistance	One of the three mildew-resistance locus (MLO) homeologs in bread wheat: TaMLO-A1 allele	Encode a protein that was shown to repress defenses against powdery mildew diseases	Particle bombardment with Cas9/gRNA expressing plasmid into immature wheat embryos // **gene knockout with Cas9/gRNA**	Yes	[6]
**Bacteria stress tolerance**						
***Crops***						
*Citrus paradisi*	Citrus canker (caused by *Xanthomonas citri* subspecies *citri* (Xcc)) tolerance	PthA4 effector binding elements (EBEs) in the Type I CsLOB1 promoter (EBE_PthA4_-CsLOBP) of the CsLOB1 (*Citrus sinensis* lateral organ boundaries) gene	CsLOB1: susceptibility gene for citrus canker CsLOB1 gene expression induced by the binding of the pathogenicity factor PthA4 to the EBE_PthA4_-CsLOBP	*Agrobacterium*-mediated transformation of epicotyl with Cas9/gRNA expression plasmid vectors // **gene knockout with Cas9/gRNA**	No	[24]
*Citrus sinensis Osbeck*	Canker resistance	CsLOB1 promoter	Susceptibility gene CsLOB1 promoter in citrus	*Agrobacterium-*mediated epicotyl transformation // **gene knockout with Cas9/gRNA**	No	[25]
*Oryza sativa*	Bacterial blight (caused by *Xanthomonas oryzae* pv*. oyzae*) tolerance	Sucrose transporter gene OsSWEET13	Disease-susceptibility gene for PthXo2 (TAL effector gene of *X. oryzae* pv. o*ryzae*)	*Agrobacterium*-mediated transformation of embryogenic callus with Cas9/gRNA expression plasmid vectors // **gene knockout with Cas9/gRNA**	No	[26]

**ABIOTIC STRESS TOLERANCE**						
**Herbicide tolerance**						
***Model plants***						
*Arabidopsis thaliana*	Cold, salt and drought stress tolerance	UDP-glycosyltransferases UGT79B2 and UGT79B3	UGT family responsible for transferring sugar moieties onto a variety of small molecules and control many metabolic processes; UGT79B2 and UGT79B3 could be induced by various abiotic stresses	*Agrobacterium*-mediated transformation with a Cas9/gRNA recombinant plasmid binary vector via floral dipping // **gene knockout with Cas9/gRNA**	No	[27]
	Glufosinate resistance and reduced trichomes formation	BAR gene GL1 gene	BAR gene confers glufosinate resistance. GL1 gene is required for trichomes formation.	*Agrobacterium*-mediated transformation with Cas9/gRNA plasmid vectors (floral dipping) // **gene knockout with Cas9/gRNA**	Yes	[28]
*Lotus japonicus*	Bioavailability of soil organic nitrogen and capability to accommodate nitrogen-fixing bacteria intracellularly to fix its own nitrogen	Single and multiple symbiotic nitrogen fixation (SNF) genes: simbiosis receptor-like kinase (SYMRK), leghemglobin loci (LjLb1, LjLb2, LjLb3)	Involved in symbiotic nitrogen fixation	*A. tumefaciens and A. rhizogenes*-mediated transformation containing the appropriate plasmids // **gene knockout with Cas9/gRNA**	No	[3]
***Crops***						
*Linum usitatissimum*	Glyphosate tolerance	5′-Enolpyruvylshikimate-3-phosphate synthase (EPSPS)	EPSPS genes encode a protein in the Shikimate pathway that participates in the biosynthesis of aromatic amino acids; EPSPS is a target for the glyphosate where it acts as a competitive inhibitor of the binding site for phosphoenolpyruvate	Protoplast transfection with ssODN and CRISPR-Cas9 plasmid // **gene replacement**	No	[15]
*Oryza sativa*	Herbicide resistance	C287 gene	The C287Tgene mutation endows rice plants with resistance to the herbicide imazamox (IMZ)	*Agrobacterium*-mediated transformation // **CRISPR-Cas9-mediated multiplex genome editing**	No	[29]
	Herbicide tolerance	Acetolactate synthase (ALS) gene	Involved in the ALS biosynthesis (amino acid biosynthesis)	Co-transformation of rice calli through particle bombardment with Cas9/gRNA expression plasmid vector and oligonucleotide donor // **gene replacement with a donor template**	No	[16]
	Glyphosate tolerance	5′-Enolpyruvylshikimate-3-phosphate synthase (EPSPS)	Involved in the biosynthesis of aromatic amino acids	Co-transformation of rice calli through particle bombardment with Cas9/gRNA expression plasmid and donor plasmid // **gene insertion and replacement with a donor template**	Yes	[30]
*Solanum tuberosum*	Reduced susceptibility to ALS-inhibiting herbicides	Acetolactate synthase 1 (ALS1)	Involved in the acetolactate synthase biosynthesis (amino acid biosynthesis)	*Agrobacterium*-mediated transformation for GVR-mediated delivery of CRISPR–Cas9 system and donor template // **gene knockout and replacement**	No	[18]
**Salt stress tolerance**						
***Crops***						
*Oryza sativa*	Salt stress tolerance	GT-1 element in the salt induction of *OsRAV2* (key regulatory regions in its promoter)	RAV subfamily involved in developmental processes such as the brassinosteroid response, leaf senescence and flowering time and also in plant responses to abiotic stress including high salinity	*Agrobacterium*-mediated transformation of leaves with Cas9gRNA plasmid expression vector // **gene knockout with Cas9/gRNA**	No	[1]
**Drought stress tolerance**						
***Crops***						
*Zea mays*	Improved grain yield under field drought stress conditions	ARGOS8	Negative regulator of ethylene responses	Co-transformation of immature embryos by particle bombardment with DNA repair template Cas9-sgRNA expression plasmids // **gene insertion or replacement with a donor template**	No	[17]

**YIELD, BIOFORTIFICATION AND CONSERVATION PARAMETERS**						
**Yield**						
***Crops***						
*Brassica oleracea* and *Hordeum vulgare*	Pod shatter and control of dormancy	HvPM19 BolC.GA4.a	Positive regulator of grain dormancy Involved in pod valve margin development	*Agrobacterium*-mediated transformation // **gene knockout with Cas9/gRNA**	Yes	[31]
*Dendrobium officinale*	Lignocellulose biosynthesis	C3H, C4H, 4CL, CCR and IRX genes	Target genes are involved in the lignocellulose biosynthesis pathway	*Agrobacterium*-mediated transformation // **gene knockout with Cas9/gRNA**	No	[32]
*Nicotiana tabacum*, *sylvestris* and *tomentosiformis*	Regulation of axillary bud growth	NtPIN4 gene	Involved in auxin biosynthesis	*Agrobacterium*-mediated transformation of leaves // **gene knockout with Cas9/gRNA**	Yes	[33]
*Oryza sativa*	Starch synthesis pathway in rice pollen	Plastific large subunit of ADP-glucose pyrophosphorylase (OsAGPL4)	Involved in the starch synthesis pathway	*Agrobacterium*-mediated transformation with Cas9/gRNA plasmid expression vector // **gene knockout with Cas9/gRNA**	No	[34]
	Regulation of pollen tube growth and integrity	Rice member of plant-specific receptor-like kinase CrRLK1LS subfamily, ruptured pollen tube (RUPO)	Receptor-like kinase (RUPO) as a regulator of high-affinity potassium transporters via phosphorylation-dependent interaction	*Agrobacterium*-mediated transformation of embryo-derived rice callus with Cas9/gRNA expression plasmids // **gene knockout with Cas9/gRNA**	No	[35]
	Grain yield performance	Grain size3 (GS3) and Grain number 1a (Gn1a)	Grain yield QTLs identified to regulate grain size and grain number	*Agrobacterium*-mediated transformation with Cas9/gRNA plasmid expression vector // **gene knockout with Cas9/gRNA**	Yes	[36]
	Grain weight	Grain width 2 (GW2), grain width 5 (GW5) and thousand-grain weight (TGW6)	Three major genes that negatively regulate rice grain weight	*Agrobacterium*-mediated transformation with Cas9/gRNAs plasmid expression vector // **CRISPR–Cas9-mediated multiplex genome editing**	Yes	[37]
	Development of japonica photo-sensitive genic male sterile rice lines	Carbon starved anther (CSA)	One important locus for regulating photoperiod-controlled male sterility in *japonica* rice	*Agrobacterium*-mediated transformation with two plasmids into calli // **gene knockout with Cas9/gRNA**	Yes	[38]
	Enhanced grain number, dense erect panicles, larger grain size	Cytokinin dehydrogenase2 (Gn1a), γ-subunit of G protein (DEP1), γ-subunit of G protein (GS3) and squamosa promoter binding protein (IPA1)	Regulators of grain number, panicle architecture, grain size and plant architecture	*Agrobacterium*-mediated transformation with Cas9/gRNA plasmid expression vectors // **gene knockout with Cas9/gRNA**	Yes	[39]
	Maintenance and determinacy of the flower meristem	FLORAL ORGAN NUMBER2 (FON2) gene OsMADS3 gene	Involved in meristem maintenance and in stamen specification	*Agrobacterium*-mediated transformation of calli // **gene knockout with Cas9/gRNA**	No	[40]
	Rice caryopsis development	OsSWEET11 gene	Sugar transporter	*Agrobacterium*-mediated transformation of leaves // **gene knockout with Cas9/gRNA**	No	[41]
	Stomatal developmental	EPFL9 gene	Positive regulator of stomatal developmental pathway	*Agrobacterium*-mediated transformation of immature embryos // **gene knockout with CRISPR–Cas9/Cpf1 system**	Yes	[42]
	Developing marker-free transgenic plants	GUS gene	Marker gene	Agrobacterium or gene gun with a construct expressing Cas9 and two gRNAs // **gene knockout with Cas9/gRNA**	No	[43]
	Rice development	MPK1 and MPK6 gnes	Essential genes for rice development	*Agrobacterium*-mediated transformation of rice calli // **gene knockout with Cas9/gRNA**	Yes	[5]
	Regulation of seed development	MEGs and PEGs genes	Involved in the regulation of nutrient metabolism and endosperm development	*Agrobacterium*-mediated transformation // **gene knockout with Cas9/gRNA**	No	[44]
	Breeding of early-maturing rice cultivars	Hd2, Hd4 and Hd5 genes	Flowering suppressors in Ehd1-dependent photoperiodic flowering pathway and major genes that negatively control the heading date of rice varieties grown in the north of China	*Agrobacterium*-mediated transformation // **gene knockout with Cas9/gRNA**	No	[45]
*Solanum lycopersicum*	Generation of parthenocarpic tomato plants	SlIAA9 gene	A key gene controlling parthenocarpy	*Agrobacterium*-mediated transformation of leaves // **gene knockout with Cas9/gRNA**	No	[46]
*Taraxacum kok-saghyz*	Rubber biosynthesis in hairy roots	TK *1-FFT* (fructan:fructan 1-fructosyltransferase)	Implicated in inulin biosynthesis (antagonist of rubber production)	TK plantlets inoculated with *Agrobacterium rhizogenes* harbouring a plasmid encoding Cas9/gRNA (wounded surface of the plantlets dipping) // **gene knockout with Cas9/gRNA**	No	[47]
*Zea mays*	High-frequency targeted mutagenesis	Argonaute 18 (ZmAgo18a and ZmAgo18b), dihydroflavonol 4-reductase or anthocyaninless genes (a1 and a4)	Involved in sporogenesis and anthocyanin biosynthesis	*Agrobacterium*-mediated transformation	No	[48]
	Reduction of the linkage drag during breeding procedure	LG1 gene	Genetic basis for the upright architecture of maize leaves	*Agrobacterium*-mediated transformation of immature embryos // **gene knockout with Cas9/gRNA**	No	[49]
**Biofortification**						
***Crops***						
*Camelina sativa*	Enhancement of seed oil (fatty acid) composition in seeds	Fatty acid desaturase 2 (FAD2) genes	Key gene involved in the synthesis of polyunsaturated fatty acids [insertion of a double bond at the delta-12 (omega-6) position of oleic acid to obtain linoleic acid]	*Agrobacterium*-mediated transformation with Cas9/gRNA plasmid vectors (floral dipping) // **gene knockout with Cas9/gRNA**	No	[50]
	Reduced levels of polyunsaturated fatty acids and increased accumulation of oleic acid in the oil	Fatty acid desaturase 2 (FAD2)	Key gene involved in the synthesis of polyunsaturated fatty acids [insertion of a double bond at the delta-12 (omega-6) position of oleic acid to obtain linoleic acid]	*Agrobacterium*-mediated transformation with Cas9/gRNA plasmid vectors (floral dipping) // **gene knockout with Cas9/gRNA**	No	[51]
	Seed oil biosynthesis	CsDGAT1 or CsPDAT1 homeologous genes	Involved in triacylglycerol (TAG) synthesis in developing seeds	Agrobacterium-mediated floral vacuum infiltration method // **CRISPR–Cas9-mediated multiplex genome editing**	No	[52]
*Hordeum vulgare* cv*.* “*Golden Promise*”	*N*-glycans modification in cereal grains	The putative endogenous barley ENGase gene	Involved in *N*-glycans biosynthesis	Co-bombarding selected combinations of sgRNA with wild-type cas9 using separate plasmids, or by co-infection with separate *Agrobacterium tumefaciens* cultures // **CRISPR–Cas9-mediated multiplex genome editing**	No	[53]
*Nicotiana tabacum*	Production of biotherapeutic proteins	XylT gene FucT gene	Involved in glycans biosynthesis	*Agrobacterium*-mediated transformation // **gene knockout with Cas9/gRNA**	No	[54]
	Production of biotherapeutic proteins	Beta(1,2)-xylosyltransferase (XylT) and alpha(1,3)fucosyltransferase (FucT).	Involved in glycans biosynthesis	Agrobacterium-mediated transformation // **CRISPR-Cas9-mediated multiplex genome editing**	No	[55]
*Oryza sativa*	Generation of high-amylose rice	SBEI and SBEIIb genes	Starch branching enzyme (SBE) genes involved in starch biosynthesis	*Agrobacterium*-mediated transformation // **gene knockout with Cas9/gRNA**	Yes	[56]
*Papaver somniferum*	Biosynthesis of Benzylisoquinoline alkaloids (BIAs): medical biomolecules	*3*′*-hydroxyl-N-methylcoclaurine 4*′*-O-methyltransferase* isoform 2 (*4*′ *OMT2*) gene	Implicated in the regulation of the biosythesis of benzylisoquinoline alkaloids (BIAs, e.g. morphine, thebaine)	*Agrobacterium*-mediated transformation of leaves with TRV-based synthetic plasmids expressing gRNA and a Cas9-encoding synthetic vector // **gene knockout with Cas9/gRNA**	No	[19]
*Solanum tuberosum*	Starch quality (amylopectin potato starch)	Three different regions of the gene encoding granule-bound starch synthase (GBSS)	Enzyme responsible for the synthesis of amylose (encoded by a single locus)	PEG-mediated protoplast transfection with CRISPR-Cas9 expression plasmid constructs // **gene knockout with Cas9/gRNA**	Yes	[12]
*Salvia miltiorrhiza*	Knock out the committed diterpene synthase gene	Diterpene synthase gene SmCPS1	Involved in tanshinone biosynthesis	*Agrobacterium rhizogenes*-mediated transformation // **gene knockout with Cas9/gRNA**	No	[57]
**Conservation parameters**						
***Crops***						
*Solanum lycopersicum*	Inhibition of tomato fruit ripening	Three regions within the RIN gene (ripening inhibitor)	Master regulator gene for tomato fruit ripening; encodes a MADS-box transcription factor regulating fruit ripening	*Agrobacterium*-mediated transformation with Cas9/sgRNA-expressing plasmid vectors // **CRISPR–Cas9-mediated multiplex genome editing**	Yes	[58]

The heritability and the transgene-free character of the generated plants were demonstrated in several studies, confirming that these areas should no longer be a concern for agricultural applications. This opens up many opportunities for different agricultural and industrial applications of CRISPR systems and below we focus on those that were developed in proof-of-concept studies.

**Figure 1.**
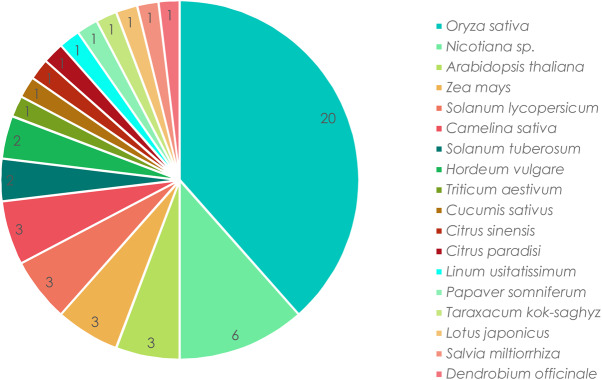
Plant species studied. Plant species studied in articles with agricultural applications (2014–2017).

## Agricultural and industrial proof-of-concept studies

To study the agricultural and industrial applications of CRISPR/Cas systems in plants, 52 articles dealing with trait improvement of crops were selected to assess how scientists are directing their use. The use of CRISPR/Cas systems covers various applications, from biotic stress tolerance to abiotic stress tolerance, and also includes the achievements of improved yield performance, biofortification and enhancement of plant quality (Table 1 and Figure 1). Table 1 summarizes the main information found in these applied research articles, with a view to

- understanding the main applications of CRISPR/Cas systems in plant genome editing;
- looking at whether the production of transgene-free plants was addressed in the studies and
- detailing the main strategy used and method of delivery of the CRISPR components.

**Figure 2.**
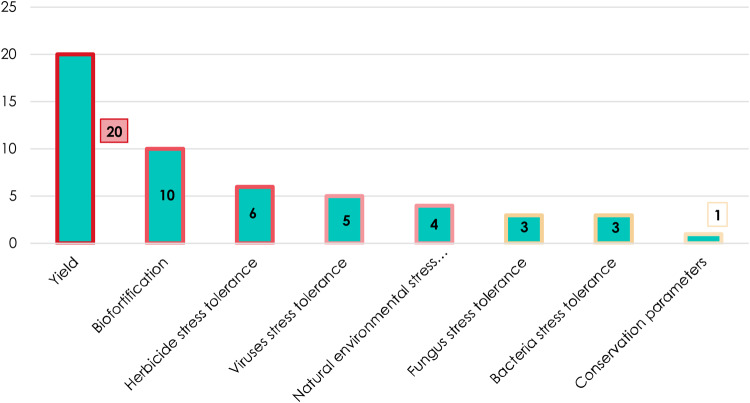
CRISPR applications. Relative importance of the different applications of CRISPR systems in terms of the number of articles (2014–2017).

First of all, the application of CRISPR/Cas systems is mainly achieved directly in crops: 42 out of 52 articles studying 15 crops (Figure 1). Few studies use model plants for transient assays before studying the stable and heritable patterns of CRISPR-induced mutations in the target crop(s). Several trends can be observed with regard to the scope of applications of CRISPR/Cas systems in plant genome editing. The most important group of target applications relates to yield traits followed by the achievement of biotic or abiotic stress tolerance (Figure 2). Biotic stress tolerance includes induced tolerance to viral, fungal and bacterial diseases with a higher number of articles exploring plant tolerances to viral disease (Figure 2). As for abiotic stress tolerance, the two main objectives are to achieve herbicide and natural environmental stress tolerances (Figure 2). Environmental stress includes cold, salt, drought and nitrogen stress. All of these trait improvements are related to economic and agronomic challenges faced by farmers as pathogens, and environmental conditions are important threats that need to be dealt with in agriculture. Furthermore, plant breeders are continually trying to increase yield performances. The most studied crop is rice (*Oryza sativa*) (Figure 1) followed by other major crops: maize (*Zea mays*), tomato (*S. lycopersicum*), potato (*Solanum tuberosum*), barley (*Hordeum vulgare*) and wheat (*Triticum aestivum*) (Figure 1). Finally, the emergence of biofortification in the list of applications can be related to that of metabolic engineering in the 1990s.

For the achievement of viral disease resistance, two main strategies are observed:

- the integration of CRISPR-coding sequence in the host plant genome that targets and interferes with the virus genome once it is incorporated in the plant: the aim is to establish a CRISPR-like immune system in the host genome [7,14] and
- the induction of a CRISPR-mediated targeted mutation in the host plant genome that will confer improved virus resistance traits [9].

As an example, Ji et al. [7] demonstrated resistance to the Geminivirus, beet severe curly top virus using a CRISPR/Cas-based approach in the model plants *Arabidopsis* and *Nicotiana benthamiana*. The resulting plants were highly resistant to the virus. An extensive knowledge of plant biology and gene functionalities is required before using CRISPR/Cas systems in a specific species for a particular application. The application of CRISPR/Cas gene editing requires the precise definition of the target DNA sequence and the availability of good genome sequence data of the studied species in order to allow design of single-guide RNAs (sgRNA). The presence of a PAM sequence (protospacer-adjacent motif) upstream of the sequence complementary to the sgRNA is also required and it is necessary to search for putative off-target sites.

Once the decision is taken to employ CRISPR/Cas systems for a given application, scientists need to choose adapted delivery methods and strategies to fulfill their objectives. Table 1 lists a selection of articles with agricultural applications that could be considered as proof-of-concept studies for future commercial application of CRISPR/Cas systems in plants. It shows that conventional *Agrobacterium*-mediated transformation using plasmid vectors containing, for example, Cas9/sgRNA expression cassettes is mainly used to deliver the system to plants. However, additional delivery methods have also been implemented such as

- protoplast transfection in *Linum usitatissimum* and *S. tuberosum* [12,15];
- biolistic delivery in *T. aestivum*, *O. sativa* and *Z. mays* [6,16,17], and
- use of reconstituted viral replicons in *N. benthamiana*, *S. tuberosum* and *Papaver somniferum* [14,18,19,59,60].

Table 1 also describes how CRISPR/Cas systems can be used not only for site-directed mutagenesis (gene knockout) but also for gene insertion or replacement and multiplex genome editing (column ‘Delivery method//Main strategy’).

## Geographic distribution of studied articles

**Figure 3.**
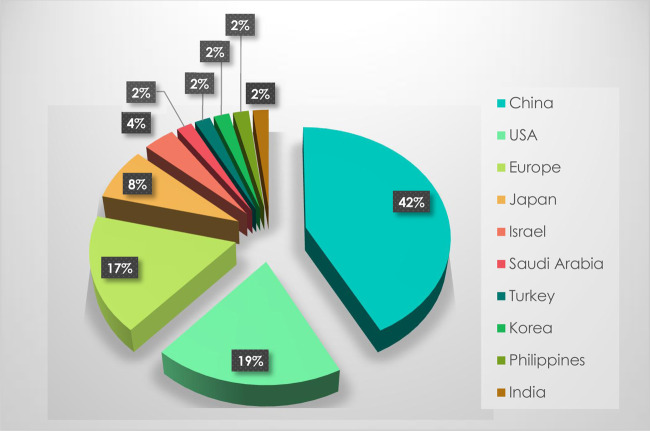
CRISPR studies by country. Number of articles studying the use of CRISPR systems in plant genome editing with agricultural applications according to the country of the research team (2014–2017).

As there are distinct research, economic and regulatory contexts in the world, it was interesting to focus on the importance of the use of CRISPR/Cas systems in plant genome editing depending on the country where studies were carried out. Regarding articles with agricultural applications, Figure 3 shows that China and the U.S.A. are ranked first with 22 (42%) and second with 10 articles (19%), respectively. Europe, which includes the U.K., Sweden, France, Hungary, Germany, Austria and Belgium, had 9 articles (17%). Four studies were carried out in Japan and two in Israel. Five studies were carried out in each of the following countries: Saudi Arabia, Turkey, Korea, Philippines and India. This figure is consistent with the globalized economic, regulatory and research contexts and can be partly explained by the uncertain regulatory framework in Europe that may be holding back work towards commercial application (Figure 3).

**Figure 4.**
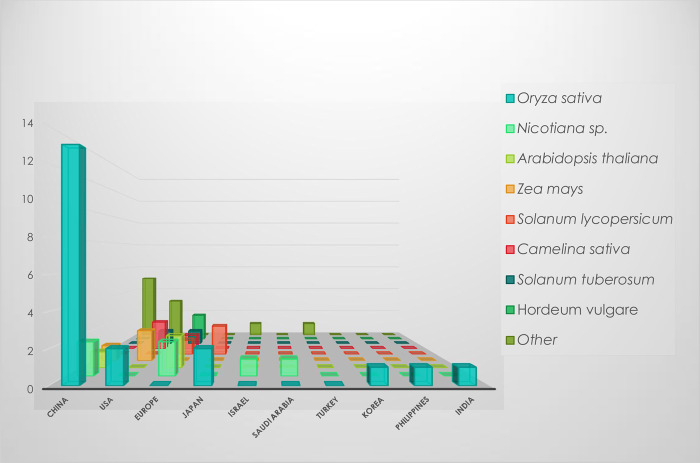
Plant species studied by country. Plant species studied in articles using CRISPR systems in plant genome editing with agricultural applications according to the country of the research team (2014–2017).

Regarding the plant species studied according to the country of the research team (Figure 4), the dominance of rice (*O. sativa*) is again observed, and mainly in China, which is in accordance with the Chinese research and economic contexts. Additionally, the application of CRISPR/Cas systems in maize (*Z. mays*) seems to be mainly studied in the U.S.A. Efficient systems for genome editing in soybean have also been reported for example [61]. Other crops that were studied include vegetables and industrial plants:

- *Cucumis sativus*, *Citrus paradisi* and *Citrus sinensis*,
- *L. usitatissimum*, *P. somniferum*, *Taraxacum kok-saghyz*, *Salvia miltiorrhiza* and *Dendrobium officinale* and
- the model plant *Lotus japonicus.*

**Figure 5.**
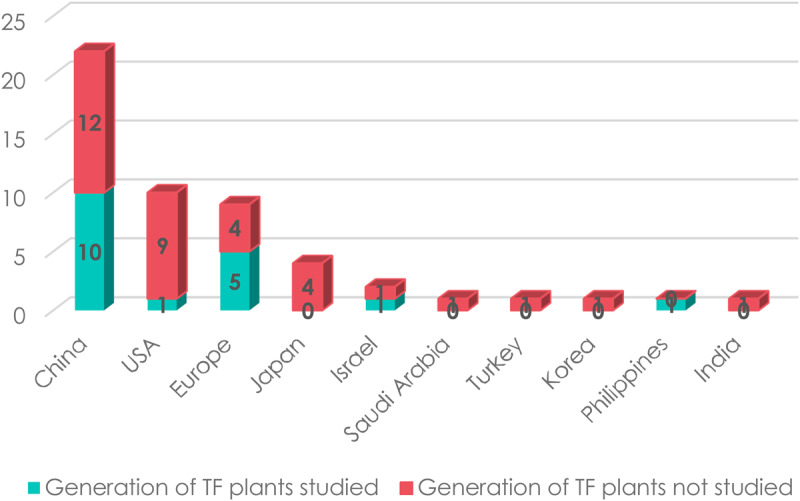
Generation of transgene-free plants by country. Sorting of the 52 articles according to the country of the research teams showing whether the generation of transgene-free plants was studied (green) or not (red).

In terms of methods, the generation of transgene-free plants (that is to say plants in which the Cas9/sgRNA-expressing sequence was not integrated) is important to examine in relation to the geographic location of the research team describing the specific agricultural application. Although the number of reviewed articles is low, one trend worth noting is that only one of the studies carried out in the U.S.A. addressed the generation of transgene-free plants (Figure 5). In contrast, Chinese and European studies paid particular attention to the generation of transgene-free plants. This is likely to be linked to GMO regulatory requirements and intellectual property considerations that differ from country to country.

## Conclusion

Since 2013, considerable progress has been made in plant genome editing thanks to CRISPR/Cas systems. This technology has allowed straightforward, cost-effective and efficient gene editing compared with previous technologies, including zinc finger nucleases (ZFNs) and transcription activator-like effector nucleases (TALENs), making it accessible to many researchers. However, this emerging method is still developing and scientific efforts must continue to be made in order to obtain a mature technology and to realize the full potential of the technology. CRISPR/Cas-based technologies are, however, advancing at a rapid pace with the description of many new technological advances. Such advances are often first described in animal systems and then transferred to plants. A recent example is the application of ‘base editing’ in a crop where a specific base change was achieved in wheat rather than the usual mutation at a specific site involving a small insertion or deletion [62].

Concerns have been raised over the relationships that may exist between the use of this method and GMOs, and the many studies related to the generation of transgene-free plants [63] show that scientists aim to demonstrate that this technology is distinct from GM technology. In the U.S.A., the legal status of CRISPR/Cas-induced mutations is that they are exempt from GMO laws. In Europe, in October 2016, the French Council of State asked the European Court of Justice whether CRISPR/Cas and other site-directed mutagenesis tools should fall under the EU GMO legislation. The European Court of Justice has 18 months to reply. Moreover, ethical concerns could also emerge regarding the impact on public health and the environment of using CRISPR/Cas in plants. However, the use of this system already represents an emerging market, with CRISPR/Cas applications spanning a wide range of industries including research, agricultural and biomedical [64]. The agricultural applications described in this literature review represent only the very first, initial uses of this exciting technology, and we can expect many more valuable opportunities for agriculture in the near future.

## Summary

- A systematic review of 52 scientific articles from 2014 to mid-2017 regarding the use of CRISPR systems for agricultural applications.
- The principal species studied is rice. The main applications are yield performance, biofortification and tolerance to abiotic and biotic stress (virus, fungi and bacteria). China published most articles in this area followed by the U.S.A. and Europe.
- The heritability of the induced mutations and the development of transgene-free plants are the most studied areas.
